# Music in the Brain: From Listening to Playing

**DOI:** 10.1155/2015/927274

**Published:** 2015-10-12

**Authors:** Masayuki Satoh, Stefan Evers, Shinichi Furuya, Kentaro Ono

**Affiliations:** ^1^Department of Dementia Prevention and Therapeutics, Graduate School of Medicine at the Mie University, 2-174 Edobashi, Tsu, Mie 514-8507, Japan; ^2^Department of Neurology, University of Münster and Department of Neurology, Krankenhaus Lindenbrunn, Lindenbrunn 1, 31863 Coppenbrügge, Germany; ^3^Department of Informatics and Communication Sciences, Sophia University, 4-4 Chiyoda-ku, Tokyo 1020081, Japan; ^4^Human Brain Research Center, Kyoto University, 54 Shogoin Kawaramachi, Sakyo-ku, Kyoto 606-8507, Japan

Music is one of the most primary abilities of human. The first case of amusia, which meant the impairment of musical ability due to the brain damage, was reported only several years after Broca's first report of aphasia. Since then, case studies were the main strategy to investigate human brain functions for a long time. Compared to remarkable progression of the study about language, music processing in the brain still remains to be clarified. But, over the last few decades, a considerable number of studies have been made on this issue, especially using the neuroimaging techniques. Now is the time to overview and integrate these findings and propose the possible application in clinical and educational situations.

The articles in this special issue include reviews and research studies focusing on cognitive, learning, developmental, and therapeutic aspects of music. The backgrounds of the authors are various: basic neuroscience, clinical neurology, pediatrics, geriatrics, and engineering. The methods include electroencephalography (EEG), positron emission tomography (PET), functional MRI (fMRI), and near infrared spectroscopy (NIRS). Based on the various points of view, the present issue shows some windows through which we can investigate the relationship between music and brain.

Several activation studies were reported using NIRS, PET, and fMRI. Mismatch Negativity (MMN) is a deviation-specific component of the auditory event-related potential (ERP), which detects a deviation between a sound and an internal representation. In the review article of “The Mismatch Negativity (MMN): An Indicator of Perception of Regularities in Music,” X. Yu et al. compared the differences of MMN features between musicians and nonmusicians, followed by a discussion of the potential roles of the training effect and the natural exposure in MMN. They pointed out some open questions and emphasized the importance to combine MMN with other experimental paradigms.

Another article related to MMN was presented by J. O'Brien et al. In the paper entitled “Interaction of Musicianship and Aging: A Comparison of Cortical Auditory Evoked Potentials,” they investigated whether the beneficial auditory neural effects of early music training persist throughout life and influence age-related changes in neurophysiological processing of sound. The results showed that MMN and P3a latencies for harmonic tone deviances were earlier for older musicians than older nonmusicians. These findings support beneficial influences of musicianship on central auditory function.

In the article “Musical Sequence Learning and EEG Correlates of Audiomotor Processing,” M. D. Schalles and J. A. Pineda studied the functional connection between motor and auditory systems during musical performance. They recorded EEG of subjects listening to clips from a song they learned to play, a transposed version of that song, and a control song with different melody and notes from the learned song. EEG power of the beta band over sensorimotor scalp showed increased suppression for the learned song, a moderate level of suppression for the transposed song, and no suppression for the control song. They interpret these findings as a support that the motor system not only was active during the covert perception of music one can play but also showed sensitivity to changes in pitch when the relative sequence of notes is preserved.

Using fMRI, K. Tabei investigated brain areas involved in perceiving and feeling emotions during music listening. In the article of “Inferior Frontal Gyrus Activation Underlies the Perception of Emotions, while Precuneus Activation Underlies the Feeling of Emotions during Music Listening,” he showed that cortical areas including the prefrontal, auditory, cingulate, and posterior parietal cortices were consistently activated by the perceived and felt emotional tasks. The precuneus showed greater activity during the felt emotion task than during a passive listening task. He suggested that the bilateral inferior frontal gyri and the precuneus are important areas for the perception of the emotional content of music as well as for the emotional response evoked in the listener.

Because of the silence during the operation, PET is suitable to study music recognition. In the PET activation study of “Sound Richness of Music Might Be Mediated by Color Perception: A PET Study” by M. Satoh et al., the cognitive processing of the perception of sound richness was investigated. The posterior portion of the inferior temporal gyrus, including the lateral occipital complex (LOC) and fusiform gyrus, was activated; so they concluded that certain association cortices may represent centers of multisensory integration in terms of both vision and audition.

The anatomical difference of the brain between musician and nonmusicians was reported in the article “A Voxel-Based Morphometry Study of the Brain of University Students Majoring in Music and Nonmusic Disciplines” by K. Sato et al. Voxel-based morphometry (VBM) uses a voxel-wise analysis method for determining focal differences in volume. They showed that the music expert group had the largest gray matter volumes in the right inferior frontal gyrus (BA 44), left middle occipital gyrus (BA 18), and bilateral lingual gyrus. These differences are considered to be caused by neuroplasticity during long and continuous musical training periods.

Functional NIRS (fNIRS) shows the activation of brain regions through decrease in oxyhemoglobin (O_2_Hb) and the increase in deoxyhemoglobin (HHb) concentration. Using fNIRS, L. Ferreri et al. aimed to extend previous findings, in the article “The Influence of Music on Prefrontal Cortex during Episodic Encoding and Retrieval of Verbal Information: A Multichannel fNIRS Study,” by monitoring the entire lateral prefrontal cortex (PFC) during both encoding and retrieval of verbal material. The results showed that music provided a less-demanding way of modulating both episodic encoding and retrieval, with a general prefrontal decreased activity under the music versus silence condition. This suggests that music-related memory processes rely on specific neural mechanisms and that music can positively influence both episodic encoding and retrieval of verbal information.

Two articles are dedicated to the research of cochlear implant recipients. Cochlear implants (CIs) are surgically implanted hearing devices that enable the perception of sound for most persons diagnosed with severe to profound deafness. In the article entitled “Melodic Contour Training and Its Effect on Speech in Noise, Consonant Discrimination, and Prosody Perception for Cochlear Implant Recipients,” C. Y. Lo et al. described the effect of two melodic contour training programs and their relative efficacy as measured on a number of speech perception tasks. Results indicated that there were some benefits for speech perception tasks for CI recipients after melodic contour training. Specifically, consonant perception in quiet and question/statement prosody was improved.

In the article of “Music Engineering as a Strategy for Enhancing Music Enjoyment in the Cochlear Implant Recipient”, G. Kohlberg et al. tested whether reengineering music to reduce its complexity could enhance the listening experience for the CI listener. As a result, compared to the original song, modified versions containing only 1–3 instruments were less enjoyable to the normal hearing (NH) listeners but more enjoyable to the CI listeners and the NH listeners with CI simulation. They concluded that, in addition to improvements in software and hardware, engineering music specifically for the CI listener may be an alternative means to enhance their listening experience.

Language and music share many properties, with a particularly strong overlap for prosody. Music perception skills (melodic and rhythmic-melodic perception and melody recognition) in a group of children with Specific Language Impairment (SLI) were shown in the article of “Music Perception Influences Language Acquisition: Melodic and Rhythmic-Melodic Perception in Children with Specific Language Impairment” written by S. Sallat et al. Children with SLI performed in most tasks below their age level, and these data strengthened the view of a strong relation between language acquisition and music processing.

Even though the accomplishment of each study is limited from the viewpoint of the field of neuroscience, we confine that the border between unknown and already known is spreading step by step. We hope that this special issue will shed light on major developments in the area of music processing in the brain and attract attention by the scientific community to pursue further investigations leading to the implementation of music in clinical, educational, and therapeutic situations.

